# Eastern Equine Encephalitis Virus Complex: Human Disease, Diagnosis and Treatment

**DOI:** 10.3390/v18070692

**Published:** 2026-06-23

**Authors:** Mohammed Umar, Evan P. Williams, Gabriel Correa Quitete Schaller Chagas, Anuj Singh, Katarina Rueda, Colleen B. Jonsson

**Affiliations:** 1 Department of Microbiology, Immunology, Biochemistry, University of Tennessee Health Science Center, Memphis, TN 38163, USA; mumar@uthsc.edu (M.U.); gchagas@uthsc.edu (G.C.Q.S.C.); anuj.singh@utrgv.edu (A.S.); krueda@uthsc.edu (K.R.); 2 College of Graduate Health Sciences, University of Tennessee Health Science Center, Memphis, TN 38163, USA; 3 Regional Biocontainment Laboratory, University of Tennessee Health Science Center, Memphis, TN 38163, USA; ewilli99@uthsc.edu; 4 Institute for Host Pathogen Systems, University of Tennessee Health Science Center, Memphis, TN 38163, USA

**Keywords:** alphavirus, Eastern equine encephalitis virus, Madariaga virus, neurological sequelae, clinical symptoms, pathology, diagnosis

## Abstract

The Eastern equine encephalitis virus (EEEV) complex comprises mosquito-borne neurotropic alphaviruses maintained in nature by mosquitoes. Although rare, human infections caused by these viruses can lead to febrile illness that may progress to severe encephalitis, for which there are no vaccines for prevention and no specific therapeutics for treatment. Moreover, a high percentage of human cases show long-term neurological sequelae. Here, we review the literature on cases, diagnosis, and management. Current gaps in clinical care include an urgent need to develop rapid diagnostic tests, new therapeutics, and vaccines.

## 1. Introduction

**Figure 1:**
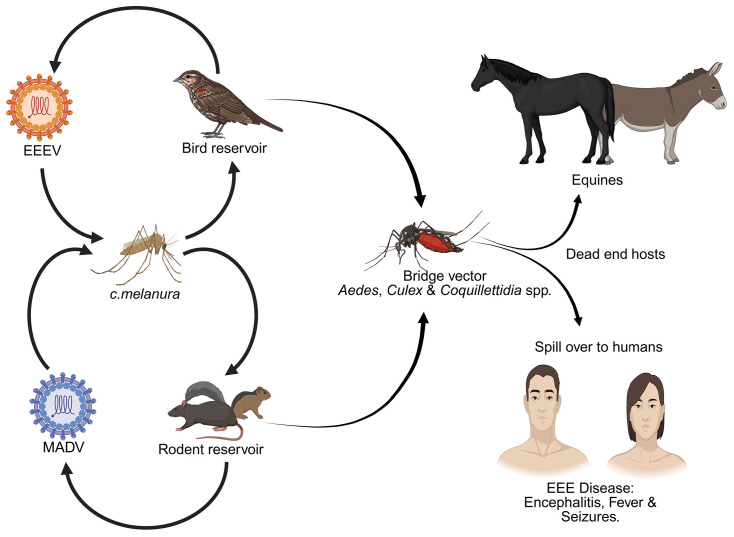
EEEV and MADV transmission cycles. Enzootic transmission cycles of Eastern equine encephalitis virus (EEEV) and Madariaga virus (MADV) occur through distinct cycles involving passerine birds and rodents, respectively, as primary reservoir hosts and *Culiseta melanura* mosquito as the principal maintenance vector. *Aedes*, *Culex*, and *Coquillettidia* spp. act as bridge vectors of EEEV and MADV to human and equine hosts, which are dead-end hosts. Infection may result in acute febrile illness and severe neurological disease, including encephalitis and seizures. Made with BioRender.

The Eastern equine encephalitis virus (EEEV) complex comprises mosquito-borne viruses with a single-stranded, positive-sense RNA genome in the genus *Alphavirus*, family *Togaviridae* [1,2]. The EEEV (lineage I) is maintained in nature in enzootic cycles between *Culiseta melanura* mosquitoes and passerine birds that serve as amplifying hosts in hardwood swamps in the eastern and southeastern coastal regions of the United States of America (USA) and Mexico [1,3]. Importantly, *C. melanura* feeds entirely on birds, meaning that transmission of the virus to humans and other dead-end mammalian hosts such as horses depends on “bridge” mosquitoes—including *Aedes*, *Coquillettidia*, and *Culex* species—that feed on both birds and mammals. The Central and South American EEEV complex lineages II-IV are distinct from EEEV and were reclassified as the Madariaga virus (MADV) [4]. While the details for the vector(s) for MADV remain under investigation, studies suggest that *Culex (Mel.) pedroi* and *Culex (Mel.) taeniopus* may serve as potential vectors [5,6]. MADV has been detected in *Oryzomys* sp., *Didelphis marsupialis* (common opossum), lizards, and bats [7,8,9]. Human infection with EEEV and MADV occurs as a result of bites from infected mosquitoes [1,2] (Figure 1).

EEEV is now recognized as one of the deadliest mosquito-borne diseases in the USA, with annual fatality rates up to 75% among individuals who develop neurological symptoms and disease [10,11,12]. Sadly, a majority of patients with EEEV-associated neurologic disease develop long-term sequelae, including seizures, altered mental status, somnolence, intellectual disability with cognitive and motor impairments [2,13,14]. These complications often lead to lifelong healthcare challenges and substantial costs, underscoring the serious impact of the disease on both public health and quality of life in the USA [11,15,16,17,18].

In Latin America, MADV is the primary agent responsible for equine outbreaks since the 1930s, but it has only rarely been linked to human disease before the outbreak in 2010 in Panama [19,20]. In this first recognized human outbreak of MADV, 53% of the cases were children and progressed to neurological disease [20]. In contrast to EEEV cases, we have limited information on neurological sequelae, and the reported fatalities are low [20,21,22,23,24,25]. Because the areas in Latin America that are endemic for MADV and the other viruses circulating, i.e., DENV, ZIKV, and CHIKV, are rural, the incidence is likely underreported [26].

Despite recent advances in research on these viruses and their diseases, supportive care remains the mainstay of management for EEEV and MADV [26,27]. Follow-up studies of survivors suggest potential for cognitive and motor recovery, with improvement seen in about 40% of patients overall and up to 75% of those with severe deficits. However, the underlying causes of these persistent sequelae remain unclear for EEEV and MADV [18,26]. The lack of commercially available rapid diagnostic tests to identify EEEV or MADV infection forces clinicians to rely on detecting viral antigen or culturing viruses from cerebrospinal fluid (CSF). This review focuses on current clinical findings from confirmed EEEV and MADV cases (Table 1), diagnostic approaches, management, and treatment, as well as limitations in these areas and potential directions for future research.

## 2. Viral Classification and Epidemiology

As mentioned previously, the species *Alphavirus eastern* (EEEV) occurs in North America and the Caribbean, while lineages 2, 3, and 4, species *Alphavirus madariaga* (MADV), circulate in Central and South America (Table 2) [52]. The genomes of the EEEV strains are highly conserved in the USA and Mexico, whereas the genomes of the Central and South American MADV strains tend to exhibit greater genetic diversity [2,4]. Because of this genetic diversity of available full-length genome sequences, Central and South American EEEV strains formerly known as South American Eastern equine encephalitis viruses were reclassified as MADV in 2010 [4]. The virus is named after the region where the virus was first isolated in 1930, the General Madariaga Partido, Buenos Aires Province, Argentina [64]. It is important to note that before the reclassification, the literature from Central and South America that references EEEV actually refers to MADV.

Confirmed cases of EEEV and MADV cases span all age groups, from 4-week-old to over 70 years old, and both sexes are represented (Table 1) [48,50]. Notably, of the 355 cases reported in the USA since 1938, the apparent average mortality is 45%, with a range of 0 to 69% by year (Table 2). An average of 11 EEEV cases are reported each year in the USA according to the CDC. Based on the available information on MADV cases, we estimate an average mortality rate of ~9% (Table 1 and Table 2). A review of the reported case studies of EEEV (Table 1) reveals that most patients were exposed to mosquitoes in rural areas while hiking, camping, hunting, or living or working in forested or bog areas [37,45,47,49,51,55,57]. Similarly, MADV cases are associated with activities in rural areas, such as agriculture, farming, cattle ranching, andfishing, inforest and swamp areas, and in housing conditions that favor mosquito breeding; for example, see [9,20,21,32,34] and other references for MADV cases in Table 1.

Since the EEE disease was first recognized in a cluster of 34 cases in the fall of 1938 in southeastern Massachusetts, cases typically occur from July through October [1,15,20,49]. The age distribution of the 34 EEEV cases reported in 1938 who were hospitalized was between the ages of one month and ten years of age, and 65% of them eventually died [65]. Additional cases in Massachusetts were not noted until 1955 and 1956, when a small cluster of four and 12 human cases were identified, respectively [30]. Since that time, EEEV cases in the USA have occurred along the Atlantic and Gulf Coasts, including New Hampshire, Florida, Louisiana, Alabama, and Texas [12,30]. Cases associated with EEEV were first reported in New Jersey in 1959, with 32 laboratory-confirmed cases over eight weeks [16,33]. More recently, in 2019, more than 70 years after the first cases reported in 1938, 38 cases with 12 deaths were reported from persons living mostly in Massachusetts and Michigan [66,67]. Interestingly, while cases in the northeastern USA tend to be seasonal—between July and October—Florida experiences cases year-round [68].

MADV had been consistently detected in equines and mosquitoes but had been reported in only three human cases in Central and South America before the 2010 outbreak in Panama. Of these three cases reported before 2010, one fatal MADV case was reported in 1955 in Brazil, and two cases, one fatal, were reported in Trinidad and Tobago in 1970 [32,34]. In the 2010 Panamanian outbreak, there were seven cases confirmed for MADV, and all survived [20]. Human and equine MADV cases began in May at the start of the rainy season. In a follow-up study of cases five years after the 2010 Panamanian outbreak, high rates of self-reported memory loss, fatigue, and a significant increase in the incidence of seizures were reported. Remarkably, none of these people had any neurological symptoms in 2010 [20]. Hence, despite the low mortality associated with the 2010 outbreak (<3%), this study suggests that even mild cases of MADV represent a serious and prolonged risk to health [21]. Lastly, cross-sectional serological studies in Panama, Peru, and Brazil report seropositivity rates to MADV ranging from 1 to 19% in human populations [9,20,69]. The high seroprevalence in Panama suggests subclinical infections in this region. A cross-sectional study of blood donors living in endemic areas conducted in 2019–2020 in the USA reported a seroprevalence to EEEV of 1.62% in one county in Massachusetts [70]. The only other study was conducted in New Jersey following an outbreak in 1959, where seroprevalence ranged from 0.9 to 6.2% [33].

## 3. Viral Life Cycle

Alphaviruses are enveloped spherical particles of approximately 70 nm with a positive-sense, single-stranded genome enclosed in an icosahedral nucleocapsid. The approximately 11,700-nucleotide genome organization and architecture are highly conserved among alphaviruses, with two open reading frames (ORFs) [71]. The first ORF encodes the nonstructural proteins (nsP1, nsP2, nsP3, and nsP4), which are essential for the assembly of the replication complex and its activity [72]. The second ORF encodes for structural proteins: the capsid, E3, E2, 6K, and E1 [73].

The proteins nsP1, nsP2, nsP3, and nsP4 support RNA synthesis [74,75]. nsP1 mediates positive-sense RNA capping of the viral genome via methyltransferase and guanylyltransferase activities [72,76]. The nsP2 provides helicase, NTPase, RTPase, and protease activities essential for RNA replication. The helicase and NTPase unwind RNA structures in concert with nsP4, while the RTPase generates the 5′-diphosphate ends required for nsP1-mediated capping [77]. The C-terminal domain of nsP2 functions as a protease that cleaves the nonstructural polyprotein in *cis* or *trans*, generating distinct replication activities during the viral life cycle [78]. While nsP3 plays a role in regulating RNA synthesis, it has not yet been visualized within the replication complex [79,80]. The RNA-dependent RNA polymerase activity is mapped to nsP4 [81,82].

The E1 and E2 proteins mediate entry by interacting with the very low-density lipoprotein receptor (VLDLR) and/or apolipoprotein E receptor 2 [73,83,84]. Various studies have shown that EEEV neurovirulence, its heparan sulfate binding phenotype, and lack of lymphotrophism are linked to three basic lysine residues, Lys71, Lys74, and Lys77 (‘‘Lys-triad’’), on the E2 ectodomain [2,85,86]. Positively charged binding sites on the EEEV E2 glycoprotein enhance interactions between EEEV-heparan sulfate and EEEV-protein receptor, suggesting these poly-functional sites increase infection in vitro and drive pathogenesis in vivo [83].

## 4. Clinical Manifestations

**Figure 2:**
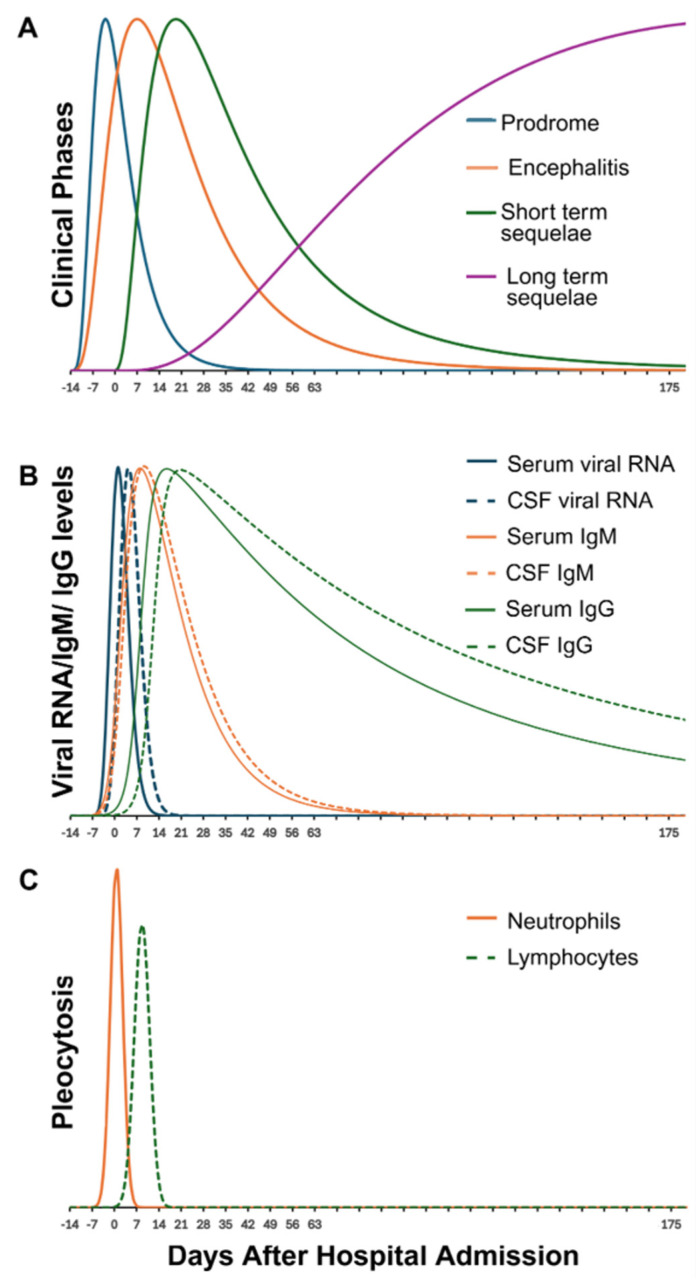
Conceptual illustration of clinical course of EEE and MADV disease. (**A**). Four major phases of disease include the prodrome, encephalitis, short-term neurological sequelae and long-term neurological sequelae. As noted in the figure, the prodrome may overlap with the encephalitic phase of disease shortly after hospitalization. (**B**). The diagram shows the general time course for the presence of virus (i.e., viral RNA) and antibodies. (**C**). Pleocytosis in the CNS is common and found early in the course of illness.

In general, clinical symptoms following infection with MADV or EEEV during the prodromal phase of illness (Figure 2A) begin abruptly with headache, high fever, and other symptoms suggestive of central nervous system involvement, such as vomiting, drowsiness or confusion, and seizures appearing within 24–48 h [10,65]. On hospitalization, patients exhibit five key symptoms that can last for over 15 days: fever, headache, nausea or vomiting, seizures, and altered mental state (confusion) [10]. Fever ranges from 38.1 °C to 40.4 °C (101 °F to 105 °F) [41,52]. In addition to fever and headaches, nausea or vomiting are some of the most common symptoms of EEEV infection [35,39,57]. EEE patients may also present on hospitalization with neck stiffness, myalgia, arthralgia, orthopnea, ataxia, malaise, facial paresthesia, and fatigue [10,35,42,49] such that the prodromal and encephalic stages of disease show overlap (Figure 2A). Short-term neurological sequelae are very common in patients, and up to 75% of EEEV survivors report long-term neurological sequelae, which include cognitive, sensory, and motor deficits (Figure 2A) [87].

Two key manifestations of encephalitis are altered mental status (i.e., consciousness, memory, recognition) and seizures (see, for example, [45,57]). Tonic–clonic seizures, recognized by loss of consciousness and violent muscle spasms, are caused by excessive, hypersynchronous neuronal discharge, possibly due to inflammation of the brain in response to EEEV infection [88]. As compared to EEE cases, the Panamanian MADV patients had a higher rate of seizures progressing to status epilepticus, which is when the patient does not regain consciousness between seizures [11,20]. EEG testing of EEEV patients shows slowing, rhythmic delta activity, and disorganization of the background EEG signal [10,35,49,89].

EEEV cases have abnormalities in the basal ganglia, thalami, cortical areas, and the brainstem as revealed by magnetic resonance imaging (MRI) and/or computed tomography (CT) [10,11,39,41,57]. Reporting on MRI is limited for MADV, but a review of four MADV case findings from the 2010 outbreak in Panama suggested that the basal ganglia and thalamic lesions, which are the predominant findings in EEE cases, were not as evident, and that other regions, such as the temporal lobes, were also involved [20,43].

In fatal cases in which an autopsy is conducted, gross examination of the brain reveals edema, leptomeningeal vascular congestion, hemorrhage, and encephalomacia [10]. The leptomeninges are membranes (pia mater and arachnoid mater) that surround the brain and spinal cord. Symptoms associated with blood accumulation in the leptomeninges include nausea, headache, and vomiting. Encephalomalacia refers to softening or loss of brain tissue, causing drowsiness and headache, and leading to permanent brain damage. Microscopic pathology includes vascular components (e.g., perivascular cuffing, small-vessel thrombosis), focal neuronal necrosis, neuronophagia, and glial proliferation and glial nodules.

## 5. Diagnostics

Early diagnosis of EEEV or MADV infections is challenging because the initial clinical symptoms are nonspecific and overlap with those of many other illnesses. The diagnosis of infection with these viruses is further hampered by the lack of widely available virus-specific diagnostic tests. The Centers for Disease Control and Prevention (CDC) has made extensive efforts since 1955 to establish diagnostic criteria for arboviruses, including EEEV [65,90] which includes virus isolation, the presence of viral antigen, or identification of IgM antibody to EEEV. Generally, blood and CSF are evaluated in microbiology laboratories for a wide range of bacterial and viral pathogens when patient infection is suspected [91]. In addition to microbiological testing, other diagnostic tests, such as blood and CSF laboratory tests, neurological imaging, and electroencephalograms (EEG), help refine the diagnosis and define disease status and progression. Each of these is reviewed in the following table (Table 3).

### 5.1. Confirmatory Microbiology

According to the CDC’s surveillance case definition for EEEV [92], a case is considered confirmed if it fulfils any one of the following: (a) the isolation of infectious virus or identification of specific viral antigen or nucleic acid from bodily fluids, tissues, or CSF; (b) there is a fourfold or greater increase in virus-specific quantitative antibody titers in paired sera; or (c) there are virus-specific IgM antibodies in serum with confirmatory virus-specific neutralizing antibodies in the same or a later specimen. A case is considered probable if serum IgM capture ELISA is positive or if a single serum sample shows high antibody titers by hemagglutination-inhibition, complement fixation, immunofluorescence, or plaque-reduction neutralization test (PRNT) [10,35,40,90].

Virus isolation is performed by amplifying the virus in mosquitoes (e.g., C6/36) or mammalian (e.g., Vero) cell lines, followed by the detection of virus antigen using RT-PCR or virus-specific serological tests [27]. Isolation of viruses from CSF or brain tissue has been successful; hence, serological testing remains the primary approach for diagnosing EEEV.

In general, IgM antibodies to EEEV and MADV can be detected in patients’ sera and CSF during the early phases of disease (Table 1). IgM antibodies have been observed as early as 1 day after symptom onset but are typically detectable within the first week [26,42,52,93]. In CSF, IgM antibodies have been reported as early as day 9 after symptom onset [94]. However, IgM antibodies can persist for an extended period (2-3 months) post-infection, so positive IgM results may represent past rather than acute infection and require additional confirmatory evidence, such as documented seroconversion in paired sera, to establish recent infection as the cause of the current illness [93]. Serological testing in immunocompromised individuals may yield false-negative results for IgM antibodies. Several case reports have documented delayed or negative serological results in patients receiving immunosuppressive therapies, which were confirmed by PCR or next-generation sequencing (NGS) [51,52,62].

In the USA, state or local health departments are contacted to arrange serologic testing in some state health departments (i.e., NYSDOH Wadsworth Center’s Laboratory of Viral Diseases) and the CDC [95]. The current Clinical Laboratory Improvement Amendments (CLIA)-certified tests available at the CDC for serum or CSF samples are an enzyme-linked immunosorbent assay (ELISA) for immunoglobulin (Ig) M and a microsphere immunoassay (MIA). The CDC also offers a CLIA-licensed plaque reduction neutralization test (PRNT). The PRNT will require biosafety level 3 (BSL-3) facilities, and the turnaround time for testing is 4 weeks according to the CDC [96]. In the older EEEV case report literature, other tests are noted, such as the complement fixation test (CF) and the hemagglutination inhibition test (HI). The CDC does not list any CLIA RT-PCR test as available.

### 5.2. Molecular Diagnostics

Molecular tests are not commercially available; however, publications suggest that nucleic acid detection through RT-PCR may provide a rapid, sensitive approach for acute infections [20,97,98,99]. The disadvantage of this diagnostic method is the limited detection window during the viremic phase (Figure 2B), which is generally between 1 and 3 days post-onset of symptoms but can be up to 7 days in some cases [22,23,25,26,57]. However, molecular diagnostic methods can distinguish between virus species, and this is critical in locations where multiple alphavirus species circulate, such as in Central and South America [20,98,99]. In the 2010 outbreak in Panama, VEEV and MADV cases were being reported, and one patient had VEEV-MADV co-infection. In the same outbreak, it was found that there was a significant amount of IgM cross-reactivity between MADV and VEEV antibodies, and hence, the PRNT, not IgM, was required for an accurate diagnosis [53]. In addition to CSF, serum, and brain samples, MADV RNA was reported in plasma and urine samples of a 12-year-old Venezuelan girl who did not develop encephalitis but had symptoms that were similar to those of dengue and Zika viruses [25]. This finding indicates that urine samples may be useful for detecting MADV/EEEV, as these viruses have a prolonged prodromal phase and IgM results may not yield definitive results in Latin American countries [100].

Metagenomics next-generation sequencing (mNGS) for the identification of arboviruses in patients has been reported [101,102,103,104], including for EEEV [62]. This approach can rapidly distinguish among multiple causative viral species, especially when their disease symptoms overlap, and could provide a turnaround time of 24–48 h.

### 5.3. Diagnostic Laboratory Tests for Blood and CSF

In addition to identification of the etiological agent, a number of diagnostic laboratory tests for blood samples are run to evaluate complete blood count (CBC) and blood chemistry and to evaluate CSF for electrolytes (e.g., sodium), cytological tests (white blood cells and red blood cell counts), glucose, and protein levels.

Hyponatremia (serum sodium < 135 mEq/L) has been reported in several EEEV case reports [10,47,60]; however, this has not been reported for any of the MADV case reports we reviewed (Table 1). In a study of 36 EEEV patients, 60% had hyponatremia, and the severity of this correlated with severe outcomes [10]. In that same study, leukocytosis was also predictive of poor outcome in 10/13 patients who had initial cerebrospinal fluid white cell counts of at least 500 cells per cubic millimeter [10]. Leukocytosis has also been reported for MADV patients [20]. Given that patients infected with either virus have elevated white blood cell counts, this stands as a critical test for these patient populations [10,20,44]. Similarly, numerous case studies have reported that EEEV patients exhibit CSF pleocytosis (Figure 2C), often with elevated neutrophil counts early in illness [10,54,57,61,62]. An increase in lymphocytic cells has also been reported [44,89]. As might be expected with high WBC counts, protein levels have been reported to be high in the CSF for some EEEV and MADV patients [10,20,44,57,89]. Lastly, case reports are not clear on glucose levels for EEEV/MADV cases, as some patients’ glucose levels were reduced [35] while others were increased in the CSF [11], but most cases have reported normal CSF glucose levels [10,11,41,46,49].

### 5.4. Neurodiagnostic Assessments

Clinical practice guidelines by the Infectious Disease Society recommend MRI in all patients with encephalitis and CT if MRI is not available [105]. Imaging using fluorine-18 fluorodeoxyglucose positron emission tomography (FDG-PET) scanning is not typically recommended [105]. Generally, neuroimaging with MRI and CT shows that EEEV and MADV cases have focal lesions in the basal ganglia, thalami, and brain stem early in the course of disease [105]. In metanalyses of EEEV patients with both CT and MRI data available, MRI demonstrates greater sensitivity, detecting more abnormalities and providing superior anatomic detail [10,42,105,106]. However, where MRI is not available, CT may help clinicians rule out other potential etiological agents, such as human herpes simplex virus encephalitis (HSE) cases, where focal low-density or hemorrhagic lesions in the frontotemporal regions are common, whereas EEE cases more often show CT findings consistent with diffuse cerebral edema [35,105]. It is important to point out that MRI will not provide a definitive etiologic agent diagnosis, as other neurotropic infectious diseases, such as West Nile virus, St. Louis encephalitis, Murray Valley encephalitis virus, and Japanese encephalitis virus, are similar to those observed in both EEEV and MADV patients [105,106,107].

A review of 36 EEE cases reported in the USA between 1988 and 1994 found that focal lesions in the basal ganglia and thalami were the most prominent on MRI in 71% of patients and on CT in 56% and 25%, respectively [10]. In this study, all comatose patients had an abnormal MRI, and hence, they suggest that a diagnosis of EEEV should be questioned if a comatose patient has a normal MRI. On MRI, T_2_-FLAIR hyperintensities (brighter areas) in EEE cases were also observed in the basal ganglia, thalami, cortical areas, and the brainstem in all patients studied [10,11,39,41,57]. These hyperintensities on MRI suggest edema, and on FLAIR, they suppress the CSF signal, highlighting this abnormality in the brain.

A review of MADV case findings from the 2010 outbreak in Panama suggested that the basal ganglia and thalamic lesions, which are the predominant findings in EEE cases, were not as evident, and that other regions, such as the temporal lobes, were involved [20]. A subsequent study of the MRI findings from one of the cases from the 2010 outbreak, a 9-year-old boy, reported T2-FLAIR hyperintense lesions in the frontal and parietal right hemisphere with bilateral basal ganglia and right thalamic-mesencephalic involvement [43].

In addition to neuroimaging, all patients with cerebral dysfunction should undergo an electroencephalogram (EEG) to identify patients with nonconvulsive seizures. Studies show that EEG findings in EEEV patients can range from normal to focal irritability and to spikes associated with nonconvulsive status epilepticus [11]. EEG results of hospitalized EEE patients often show generalized slowing, rhythmic delta activity, and disorganization of the background EEG signal [10,35,49,89]. Notably, some EEE patients have exhibited periodic lateralizing epileptiform discharges on EEG, a pattern characteristic of HSE [10]. Currently, MADV case reports lack EEG findings in patients despite seizures being common. EEG does not confirm the etiologic agent of the infection [11,105]. Improvement of EEG during hospitalization is suggested to correlate with a good prognosis for patients with viral encephalitis, but severe symptoms do not correlate with the disease [105].

## 6. Treatment and Management

There are no specific therapeutics or antivirals available for treatment of EEE or MADV cases. Our review of the literature suggests that the major approaches to treatment of EEE or MADV are empiric antimicrobial therapy, immunomodulatory interventions, antiseizure medications, and supportive intensive care. We review each of these areas reported in these papers in the following with the caveat that the details of supportive care are often not well defined or in some cases discussed.

In the majority of case reports (Table 3), antibiotics were administered on the day of hospitalization in case of suspected bacterial encephalitis (77%), with ceftriaxone (64%) and vancomycin (43%) commonly reported [37,38,41,42,44,46,47,54,55,56,58,61,62,108]. The antiviral acyclovir was used in 64% of the case reports reviewed (Table 3), and it was typically used at or near the start of hospitalization before etiologic confirmation of herpes virus, as recommended [37,38,41,42,44,46,47,54,55,56,61,108]. Early treatment is critical for the effective treatment of herpes viruses, and therefore, it is recommended that it be initiated in all patients with suspected encephalitis as soon as possible, pending diagnostic results [105,106]. In summary, the majority of case reports suggest that antibiotics and acyclovir were initiated together during hospitalization (Table 4).

Corticosteroids and intravenous immunoglobulin (IVIG) were commonly reported as immunomodulatory interventions. Anti-inflammatory medications such as dexamethasone, prednisolone, and methylprednisolone were administered in 55% of case reports and were given at various times during hospitalization depending on disease progression [10,20,37,38,41,44,47,52,56,58,62,108]. IVIG was administered in combination with high-dose methylprednisolone in four of the seven case reports that reported using IVIG [38,44,52,58,62,108].

Antiseizure medications were administered in 46% of the case reports reviewed (Table 4). Lorazepam and levetiracetam, or the combination of the two, were administered in four and seven case reports, respectively [56,58,60,62]. Other antiseizure treatments, such as phenytoin, pentobarbital, and valproic acid, were administered in two case reports each [44,46,55]. About half of patients with EEE and a significant amount of MADV patients experience seizures which were nonconvulsive status epilepticus [20,44,55,109]. This type of seizure is often managed by anti-epileptic drugs, including levetiracetam, valproic acid, and lacosamide, along with continuous EEG monitoring [55,58,62]. In other cases, the seizures were focal tonic seizures with secondary generalized convulsions [10,48,49,62], which are typically managed in the hospital through careful titration of multiple anti-epileptic medications, such as phenytoin, levetiracetam, valproate, phenobarbital, and propofol, along with continuous EEG monitoring to guide therapy and asses seizure control [44,49,55,58,62]. Sedatives and narcotic agents have been administered as supportive therapy to mitigate the excessive ventilatory drive observed in one patient with neurological involvement who exhibited persistent tachypnea despite adequate ventilation [40]. In a second case, propofol and fentanyl were used to sedate the patient [44]. Sedatives are also often used in patients on mechanical ventilation [44,49,58,62], which will be discussed briefly in the following.

Mechanical interventions, such as airway support, temperature management, and intracranial pressure monitoring, are common in management of EEE and MADV cases. In some patients who have acute hypoxemic respiratory failure, mechanical interventions are needed to support adequate gas exchange to minimize harm to the lungs; for example, see [40,51,60,62]. Intubation is commonly employed to enable adequate respiration; for example, see [47,49,55,58]. In one case report, passive cooling along with antipyretics was used, and a prophylactic catheter was placed to alleviate the patient’s pain and support ongoing supportive care management [40]. In a second case, intravenous fluids, intubation, and the use of cooling blankets (an Arctic Sun device) were used to reduce the patient’s core temperature [61]. Lastly, intracranial pressure monitoring is often used in management of encephalitis and usually in conjunction with other interventions. For example in one case, interventions included intubation for airway protection, use of an intracranial pressure monitor, placement of an external ventricular device, and plasma exchange [58]. The diuretic mannitol, with 3% sodium chloride, was administered in two case reports to reduce intracranial hypertension [44,46]. Despite hyponatremia being commonly reported in EEE cases (Table 1), reports of only two severe cases discussed management with hypertonic saline [58].

## 7. Future Directions and Research Gaps

Although reported EEE and MADV case numbers in the USA and Latin America are relatively low [33,69], the overall public health burden is substantial given their morbidity and mortality. This is largely because there are no effective vaccines to prevent or treatments to limit brain infection and neurological damage or to promote recovery for those with short- or long-term neurological sequelae [16,87]. Hence, future research that brings to market vaccines for those living in endemic areas and therapeutics for treatment would have a substantial impact. Secondly, disease management could be improved through rapid diagnostic testing. Potential research directions and gaps in these areas are addressed below; however, it is important to note that effective use of direct-acting viral therapeutics requires accurate and timely diagnostics.

Vaccines for disease prevention would benefit those living in endemic regions [110,111,112]. Between 2008 and 2025, five clinical trials in Phase 1 or 2 have been reported for EEEV at ClinicalTrials.gov [111] but none for MADV. All have been completed, except for a Bavarian Nordic Phase 2 Trial (NCT06899802) in healthy adults to test a recombinant MVA-BN-WEV protein-based vaccine with structural proteins E1, E2, E3, and 6K for VEEV, EEEV, and WEEV [113]. The Phase 1 trial of MVA-BN-WEV was completed in 2020, and results showed that the candidate was safe and that the treatment resulted in the production of neutralizing antibodies against all vaccine targets [113]. The safety profile in the Phase 1 clinical trial has led to the initiation of Phase 2 in 2025, which is active, not recruiting. In the three other clinical trials, two other platforms were evaluated: (1) TSI-GSD 104 [114], a formalin-inactivated dried EEEV; and (2) VRC 313, a trivalent virus-like particle of VEEV, EEEV, and WEEV. TSI-GSD 104 completed two Phase 2 trials in 2017 and 2018. TSI-GSD 104 was safe and immunogenic; however, vaccination is limited to at-risk laboratory personnel, and there is currently no plan to license this vaccine [114]. During the Phase 1 assessment, three individuals at the U.S. Army Medical Research Institute of Infectious Diseases were exposed to EEEV and were protected, suggesting the vaccine’s efficacy. VRC 313 completed its Phase 1 trial in 2020, and the results indicated that it was safe and well tolerated and that neutralizing antibodies against all vaccine targets were produced. To our knowledge, mRNA vaccine platforms have not yet been reported for these viruses, and given their success with other viruses, research in this area would be value-added.

Antivirals for the treatment of the encephalitic alphaviruses would be of great benefit, as most patients experience neurological symptoms shortly after admission. While several small molecules have been reported for the encephalitic alphaviruses, none have progressed into clinical trials [115,116,117]. Given the large incidence of neurological symptoms, it will be essential to discover and develop new antivirals that cross the blood–brain barrier. Another important research gap is the lack of host-targeting therapeutics to treat the resulting immunopathogenesis of infection or those host responses that support replication [117]. Certainly, future research would benefit from the discovery of small molecules that inhibit replication of VEEV, EEEV, and WEEV, whether they are host-directed or directly acting against the virus itself.

As with most human RNA viruses, rapid diagnostic tests and treatments are not available commercially, and diagnostic testing for EEEV in the USA is limited to only a few states and CDC laboratories. In the USA, EEEV is primarily diagnosed by IgM testing followed by PRNT, which has a 4-week turnaround time [11,105]. Similar approaches are used in the confirmatory diagnosis of the MADV in Latin America, which also faces additional challenges with the co-circulation of VEEV [98,118,119,120,121]. As discussed in several case reviews, early IgM testing may result in false negatives or positives, so molecular tests that improve early detection and identification of these pathogens would support care and help rule out other potential causes and unnecessary empiric treatments (e.g., acyclovir, antibiotics). Quantitative RT-PCR of blood for the virus remains challenging given the short viremic window; however, inclusion of the virus in a diagnostic panel that multiplexes for all possible etiologic agents of encephalitis would be a significant technological advance. A retrospective study of diagnostic methods for MADV in Panama from 1961 to 2023 reported that infections were identified (n = 26 [92%]) by ELISA IgM, except for one case, where RT-PCR confirmed the presence of viral RNA in autopsy brain samples [26]. Hence, the utility of the RT-PCR approach as a standard approach is unclear and requires further research. The development of new diagnostic approaches that use metagenomic or targeted NGS of the CSF may provide the sensitivity required to detect these viruses in this fluid. However, given the success of antibody-based diagnostics, lateral flow tests that detect IgM antibodies or viral antigens may represent an improved approach. This type of test would also have obvious advantages in rural health care settings where the vast majority of these cases occur.

In addition to improving the speed and sensitivity of current diagnostic testing, this review highlights the importance of implementing laboratory tests for hyponatremia and leukocytosis in suspected cases of encephalitis, both of which correlate with poor outcomes. Generally, case reports and reviews would benefit from including additional information on the symptoms and treatment regimens employed, as well as their outcomes. Lastly, there are no published studies that follow up on patients with long-term neurological sequelae to inform the effectiveness of treatments employed.

## 8. Conclusions

In summary, EEEV infection can result in severe disease with high mortality that typically results in neurological disease, and while studies are limited, many of these cases are associated with long-lasting neurological sequelae. Similarly, MADV causes severe disease, although the mortality is lower but still much higher than most viral infectious diseases. Even less is known about the neurological sequelae in MADV patients. Although no FDA-approved antivirals or vaccines are available yet for the treatment of EEEV or MADV, once antivirals are available, rapid viral-specific diagnosis will be critical given the disease’s rapid progression in the brain. The evaluation of these countermeasures is complicated by the low incidence of these diseases, which renders human clinical trials challenging, and by the overlap in virus species causing encephalitis across regions in Central and South America. The use of animal models might be considered for the efficacy testing and approval of vaccines and treatments under the FDA’s Animal Rule. Animal models will also benefit from the translation of clinical insights from studies of human disease progression and neurological sequelae.

## Figures and Tables

**Table 1:** Major clinical findings and diagnostic indicators reported in selected human EEEV and MADV case reports.

			Clinical Course		Diagnosis		
Year	Country	Age	Clinical Findings	Outcomes	Neuroimaging	Viral Diagnostic Tests	Ref.
1938 –1956	USA	0–59 yr	In Massachusetts, 50 EEEV cases were reported. Infants exhibited a sudden onset of fever, irritability or drowsiness, cyanosis, and severe convulsions. Older patients had a slower onset featuring frontal headache and dizziness and progressed to neck rigidity, continuous tremors, muscular twitching, and coma.	68% (34/50) were fatal, and survivors developed severe, long-term neurological disability. Autopsy found severe edema and congestion of the brain and cord, with maximum changes in the brain stem. Some survivors were expected to continue with permanent mental changes.	Not reported.	EEEV. Neutralizing antibodies assays on blood and virus isolation brain	[15,28,29,30]
1948–1949	Dominican Republic	10 mo–20 yr	The outbreak mainly affected children in the Province of Monte Cristi, with clinical manifestations including fever, vomiting, and generalized malaise. By the second or third day, severe convulsions appeared.	69% (9/13) were fatal within 3–8 days, and the survivors developed severe spastic paralysis.	Not reported.	EEEV-positive sera neutralization tests	[31]
1955	Brazil	2 yr	Female patient from Bahia who, before showing nervous manifestations, presented with “flu” symptoms, including coughing and sneezing. Fifteen days later, she had a high fever, followed by tonic convulsions, with clonic contractions of the right hand and deviation of the head and eyes to the right.	The patient became nonresponsive and died.	Not reported.	MADV-positive virus isolation from brain, positive PRNT	[32]
1959	USA	0–65 yr	Outbreak in New Jersey of 32 cases. Limited information was provided; however, patients were diagnosed with purulent meningitis, bulbar poliomyelitis, polioencephalitis, and general encephalitis.	69% (22/32) were fatal. Fatality was higher in the older adults and children (5–14 yr). Neurological sequelae were noted in survivors.	Not reported.	EEEV-positive serology and/or virus isolation	[33]
1970	Trinidad, Tobago	58 & 38 yr	A 58-year-old patient presented with fever, headache, diminished reflexes, neck stiffness, and a positive Kernig’s sign and entered into a coma. A 38-year-old patient had headache, fever, malaise, epileptiform convulsions, loss of consciousness, and coma.	Patient (58 yr) died four days after admission. Autopsy of the brain revealed hemorrhage, edema, and neuronal necrosis. Patient (38 yr) recovered after 14 days in hospital without sequelae.	Not reported.	MADV-positive virus isolation, positive HI, CF, neutralization	[34]
1970–1984	USA	0–69 yr	16 cases were reported. Patients had severe headaches, high fevers, and profound alterations in consciousness, such as somnolence and confusion. Physical and neurological examinations frequently revealed a stiff neck, lethargy, and vomiting.	31% fatality rate in MA, with fatality in cases ages >50 (4/6). Patients under 10 years of age experienced severe neurological sequelae (4/5), with 1 fatality out of 16.	CT revealed edema in 4/10 patients.	EEEV IgM-positive serum by CF, PRNT, and positive virus isolation from the brain	[35]
1980	USA	13 yr	A male patient from southwestern Michigan presented with fever and seizures. CSF had pleocytosis.	Patient recovered.	CT was normal.	EEEV-positive neutralization test	[36]
1992	USA	59 yr	A man from Massachusetts presented with a transient headache (prolonged prodrome), lethargy, fever, and nausea. He progressed to have left-sided hemiparesis and entered into a coma.	The patient had a complete recovery.	MRI showed lesions in the thalami and midbrain. CT suggested basal ganglia changes.	EEEV IgM- and IgG-positive serum and CSF	[37]
1988–1994	USA	6 mo–85 yr	36 cases from eleven states, with fever, headache, nausea, vomiting; 97% CSF pleocytosis/high protein/leukocytosis (69%), hyponatremia (60%).	36% mortality rate, with another 35% having moderate to severe impairments, with only one full recovery.	MRI/CT revealed lesions in the basal ganglia, thalami, and brain stem (less visible on CT).	EEEV IgM-positive serum/CSF	[10]
2001	USA	69 yr	A male from Massachusetts presented with headache, fever, and mild upper extremity weakness. Patient progressed to mild hyponatremia, lower extremity flaccidity, diffuse hyperreflexia, and bilateral Babinski signs.	The patient was discharged after 10 weeks with mild cognitive issues which resolved over the following year.	MRI showed T2 hyperintensity in the brainstem, cervical cord, tectal plate, and periventricular white matter.	EEEV IgM-positive CSF and serum	[38]
2003	USA	8 yr	A female presented with a sore throat and fever and was unresponsive; condition progressed to severe seizures.	The patient recovered from seizures within 2 weeks and was discharged to a rehabilitation facility.	MRI and CT revealed abnormalities in the basal ganglia. EEG showed bilateral hemispheric dysfunction.	EEEV IgM- and IgG-positive serum	[39]
2007	USA	51 yr	African-American man presented with an atypical array of symptoms such as dysphagia, odynophagia, persistent fever, nausea, pedal edema, orthopnea, paroxysmal nocturnal dyspnea, and myocarditis. Patient progressed to neurological symptoms and an altered mental state.	The patient developed multi-organ failure and died.	CT was unremarkable. MRI showed T2 prolongation in the bilateral thalami and basal ganglia and in the right posterior parietal cortex.	EEEV-positive IgM and IgG serum; PRNT	[40]
2007–2008	UK	35 yr	A UK man from Edinburgh who visited Rhode Island in the USA presented with fever, headache, and tonic–clonic seizures, which progressed to dysarthria, confusion, and coma for 4 weeks.	At 6 months after the coma, the patient was severely disabled with spastic quadriparesis.	CT was normal. MRI T2 intensities were found in the medial temporal lobes, basal ganglia, and brainstem.	EEEV-positive IgM and IgG in serum and CSF, positive PRNT	[41]
2007–2008	USA	5 mo	First case of EEEV-associated hemophagocytic lymphohistiocytosis (HLH) in an infant. He presented with fever, seizures, lethargy, decreased appetite, and a bulging anterior fontanelle, hepatosplenomegaly, and pancytopenia. He was intubated and mechanically ventilated.	The patient’s neurological status continued to deteriorate, and he subsequently died after 6 weeks. The brain biopsy revealed necrosis and chronic inflammation consistent with chronic encephalitis.	MRI revealed extensive cystic-type changes in the grey matter.	EEEV-positive serum and CSF IgM and IgG	[42]
2010	Panama	1–10 yr	Patients had fever, headache, vomiting, and diarrhea; 6/7 cases had seizures that evolved to status epilepticus. Additional evaluation of patients who did not meet the case definitions or were suspected of meeting them identified 6 additional cases.	Survivors were followed for 5 years, and some had neurological symptoms such as severe memory loss, dizziness, chronic fatigue, difficulty concentrating, confusion, depression, and irritability.	In the case report of a 9-year-old patient, MRI showed multiple hyperintense areas in the right frontal and parietal hemispheres and in the bilateral basal ganglia and thalamic regions.	MADV IgM- and PRNT-positive	[20,43]
2010	USA	21 yr	A male presented with headache, fever, and vomiting. Examination of the patient found hyponatremia, leukocytosis, cerebral edema, and hypertension. CSF had pleocytosis.	The patient continued to have cognitive and memory deficits after 1 year.	CT and MRI showed abnormalities. EEG showed recurrent seizure activity.	EEEV IgM-positive serum and CSF	[44]
2012	USA	39 yr	A male veterinary technician from the southeastern USA presented with fever, headache, and photophobia. The patient fell into a coma for 2 weeks. CSF had significant pleocytosis.	The patient was discharged to a nursing facility in a vegetative state.	MRI showed hyperintense signals in the basal ganglia and midbrain.	EEEV IgG- and IgM-positive CSF	[45]
2013	USA	13 yr	A male from southwestern Arkansas presented with headache, seizures, fever, tachycardia, hypotension, and raised intracranial pressure.	The patient died on day 19 after symptom onset.	MRI on the 4th day of onset showed hyperintense signals in the basal ganglia and midbrain. CT revealed frontal and temporal cerebral edema on day 6 of onset.	EEEV CSF IgM-positive by IFA and PRNT, IgM- and PRNT-positive serum	[46]
2014	USA	40 yr	A male presented with fever, throbbing occipital headache, neck stiffness, jaw pain, hyponatremia, loss of right temporal gaze, and depressed mental state. He became unresponsive, developed tachypnea, and lost his right temporal gaze, as well as his cough and gag reflexes.	The patient died on day 5 of admission.	MRI revealed abnormalities of the cortex, deep gray matter, and brainstem. EEG showed a moderate amount of cerebral dysfunction on day 3.	EEEV IHC- and PCR-positive brain tissue	[47]
2014	USA	1 mo, 7 yr	Both male patients had fever and lassitude.	Both patients died.	Not reported.	EEEV-positive HI and neutralization titers in serum	[48]
2015	USA	50 yr	A male from Florida presented with fever and progressed to an altered mental status, tonic–clonic seizures, and urinary incontinence.	Patient recovered with mild memory impairment.	MRI showed abnormal hippocampi and left frontal and parietal regions. EEG showed moderate slowing but no seizure activity.	EEEV IgM-positive serum	[49]
2015	Panama	NA	Five cases in Panama presented with fever, headache, seizures, and nausea.	Patients recovered.	Not reported.	MADV ELISA IgM-positive	[26]
2015	Brazil	10 yr	A boy from Cuiaba, Brazil, presented with acute febrile symptoms without neurological signs.	Patient recovered.	Not reported.	MADV RT-PCR-positive	[23]
2016	USA	>70 yr	An elderly male presented with fever, left arm weakness, vomiting, and headache. The patient had a moderate CSF pleocytosis. This is the first reported case with eye involvement with a progressive neurological impairment and with retinal and optic nerve inflammation.	The patient died from respiratory failure seven days after admission.	CT was unremarkable on admission but showed an infarction on day 2. MRI on day 2 suggested meningoencephalitis. EEG suggested seizure activity.	EEEV ELISA IgM-positive in serum (not CSF) and histopathology of the retina	[50]
2015–2016	Haiti	3–12 yr	Eight school children presented with fever, cough (5/8), headache (4/8), and (3/8) nonspecific abdominal pain. Only one patient presented with confusion and seizures, but this patient had a seizure history.	All the children recovered without apparent sequelae.	Not reported.	MADV isolation (plasma), RT-PCR-positive, and Sanger sequencing confirmation	[22]
2016	Venezuela	12 yr	A Venezuelan girl with an abrupt onset of weakness, generalized malaise, headache, and nausea that progressed to a high-grade fever lasting two days, which was followed by severe fatigue.	Patient recovered.	Not reported.	MADV plasma vRNA PCR-positive and Sanger sequencing	[25]
2016	Panama	NA	Three cases presented with fever, headache, seizures, and nausea.	Patients recovered.	Not reported,	MADV ELISA IgM-positive	[26]
2017	USA	55 yr	A male from New Jersey presented with fever, vomiting, headache, and normal mental status. He developed left gaze preference, left arm weakness, and coma requiring ventilatory support.	The patient died. Brain autopsy revealed patchy necrosis in the substantia nigra, basal ganglia, basal forebrain, thalamus, cerebellar, and dentate nucleus and also in the region of the claustrum and external capsule.	CT was normal. MRI showed abnormality within the basal ganglia, thalamus, midbrain, and pons.	EEEV brain biopsy PCR-positive	[51]
2017	USA	40–50 yr	EEEV transmission from a female organ donor to three female solid organ transplant recipients who developed fever, headache, hypotension, altered mental state, and left-sided gaze preference.	100% fatal. All three patients died 3 to 4 months after transplant.	MRI conducted 7 to 12 days after transplant showed multiple lesions in the brain in the basal ganglia, thalami, and brainstem.	EEEV ELISA IgM-positive in serum and CSF, PRNT, RT-PCR	[52]
2017	Panama	NA	Seven cases of MADV presented with fever, headache, seizures, and nausea.	14% fatality (1/7).	Not reported.	MADV ELISA IgM-positive, RT-PCR	[21,26,53]
2018	USA	3 mo	A male infant from Louisiana presented with fever, nasal congestion, and abrupt seizure-like activity. CSF showed pleocytosis and elevated spinal protein.	The patient suffered severe neurological sequelae.	CT was unremarkable. EEG revealed severe diffuse cerebral dysfunction.	EEEV-positive IgM antibodies in serum; negative CSF	[54]
2018	USA	57 yr	A woman from Florida presented with flu-like symptoms, lethargy, and impaired concentration that led to coma.	Patient recovered from a comatose state but developed aphasia and paralysis.	CT was normal. MRI showed intensity in the striatum. EEG suggested non-convulsive epilepsy.	EEEV IgM-positive serum, California serogroup encephalitis virus IgG positivity	[55]
2019	USA	42 yr	A male from New Jersey presented with intractable headache, facial paresthesia, and malaise. On day 2, he had a high fever, and on day 3, seizures began with altered consciousness. The patient required 9 days of mechanical ventilation.	The patient continued to suffer from moderate aphasia.	MRI revealed edema and abnormal signal in the basal ganglia to the midbrain.	EEEV IgM- and IgG-positive CSF	[56]
2019	USA	54–78 yr	Eight cases from Michigan during August showed fever, confusion, generalized weakness, seizure (2 cases), and abnormal neurological symptoms. CSF pleocytosis progressed to a lymphocytic state.	Six patients died. The two survivors had mild and severe neurological sequelae, respectively.	MRI revealed abnormalities in the basal ganglia and thalami. CT only showed hypoattenuation.	EEEV IgM and IgG antibodies in CSF; RT-PCR (CSF), PRNT (blood). One case cross-reacted with the La Crosse virus	[57]
2019	USA	6–58 yr	4 cases—3 male (ages 50, 57, 58) and 1 female (age 6)—in southern New Hampshire presented with headache and fever. Fatal cases progressed to seizure and altered mental status and then progressed to hyponatremia and coma.	50% (2/4) fatality.	MRI revealed abnormal basal ganglia, thalami, and brainstem in severe cases, but abnormalities were mild or absent in survivors.	EEEV IgM- and PRNT-positive in CSF or serum	[58]
2019	Panama	1 mo to 2 yr 11 mo (range)	3 cases presented with fever, headache, seizures, and nausea.	Two patients recovered and one died.	EEG showed cerebral dysfunction in all cases.	MADV ELISA IgM-positive	[26,59]
2020	USA	64 yr	A female from Nevada with nonspecific illness for 3 weeks presented with hyponatremia, tonic–clonic seizures, and bilateral Babinski response.	Patient died on day 24. Gross findings on necropsy were normal. Microscopic pathology showed necrosis with neuronal loss throughout the brainstem and thalamus, with associated macrophage infiltrates and reactive astrogliosis.	MRI revealed abnormal signal in the basal ganglia and thalami. EEG showed nonconvulsive status epilepticus.	EEEV IgM-positive serum and CSF	[60]
2021	USA	61 yr	A female from Georgia presented with dysarthria and a right-sided facial droop. The patient developed a fever and altered mental status and became comatose. Serum leukocytosis and CSF pleocytosis were noted.	The patient died from acute respiratory failure and meningoencephalitis.	CT and MRI were unremarkable. EEG suggested severe encephalopathy.	EEEV IgM-positive CSF;	[61]
2022	USA	27 yr	An immunocompromised man from Ohio presented with nausea, vomiting, fatigue, and fever. The patient had CSF pleocytosis. Patient’s neurological status deteriorated with loss of pupillary and corneal reflexes.	The patient died 9 days after admission.	MRI showed bilateral thalamic and basal ganglia T2 hyperintensities. EEG revealed infrequent seizure activity. CT was normal.	EEEV-positive with metagenomic NGS of CSF	[62]
2023	Panama	NA	One case presented with fever, headache, seizures, and nausea, and one case was asymptomatic.	The patient recovered.	Not reported.	MADV RT-PCR-positive	[26]
2023–2025	USA	NA	29 confirmed cases reported to the CDC in 2023–2025. No published case reports have been reported through March 2026.	24% fatality (7/29).	NA.	EEEV	[63]

Abbreviations: CF, complement fixation test; CT, computer tomography scan; CSF, cerebrospinal fluid; EEG, electroencephalogram; ELISA, enzyme-linked immunosorbent assay; HI, hemagglutination inhibition test; HLH, hemophagocytic lymphohistiocytosis; IgM, immunoglobulin M; IgG, immunoglobulin G; IFA, immunofluorescence assay; IHC, immunohistochemistry; MRI, magnetic resonance imaging; NA, not available; NGS, next-generation sequencing; PCR, polymerase chain reaction; PRNT, plaque reduction neutralization test; RT-PCR, reverse transcription PCR; vRNA, viral RNA.

**Table 2:** General comparison of EEEV and MADV.

Virus	Mortality	Genetic Diversity	Neuroimaging	Epidemiology
*Alphavirus eastern* EEEV	~45%	Low, <3% nucleotide divergence among isolates	MRI: ↑T2/FLAIR signal in frontal cortex, basal ganglia (lentiform/caudate), thalami, and midbrain; ±internal/external capsule in CT: hypodensities in basal ganglia (putamen/caudate), thalamus, and brainstem; ± cortical involvement.	Increased incidence in males, children, and older adults. Occurs in northeastern, upper mid-Atlantic, and Great Lakes USA and Mexico. Transmitted primarily by *Culiseta melanura*.
*Alphavirus madariaga,* MADV	~8.7%	17–21% nucleotide; 3–5% amino acid divergence among isolates	Limited data; MRI: Single pediatric case—↑T2/FLAIR lesions in thalamo-mesencephalic and right subcortical frontoparietal regions.	Predominantly affects children and young adults. Occurs in the Caribbean (Haiti), Central and South America. Transmitted by *Culex (Melanoconion) taeniopus/pedroi* mosquitoes.

**Table 3:** Commonly used tests in diagnosis of EEEV and MADV cases.

Test Category	Diagnostic Method	Purpose/Use
Confirmatory microbiology	Virus isolation	Confirm the presence of infectious virus in brain and CSF.
	ELISA IgM	Confirm presence of viral antigen of antibodies to virus in serum or CSF.
	PRNT	Confirm the species of virus isolated from tissues.
	IFA	Confirm the presence of viral antigen in tissue biopsy.
Molecular diagnostics	RT-PCR	Confirm the identity of viral RNA in blood or CSF or brain.
	Metagenomics	Confirm the genetic sequence of the virus.
Laboratory and blood tests	Cytological tests	Identify the levels of immune cells in CSF.
	Sodium levels	Determine if patient has hyponatremia.
	Glucose levels	Determine if patient has normal glucose levels. High glucose is typically associated with bacterial meningitis.
	Protein level	High protein levels are common in EEEV patients.
Neurodiagnostic tests	MRI	Determine if the brain is affected by infection by associated abnormalities such as edema and hemorrhage.
	CT	Determine if the brain is affected by infection by associated abnormalities.
	EEG	Determine if the electric signal in the brain has any abnormalities.

**Table 4:** Treatment for the management of EEEV/MADV disease.

Antibiotics		Acyclovir		Anti-Inflammatory		Anti-Seizure		IVIG		
Drug	Admin	Drug	Admin	Drug	Admin	Drug	Admin	Drug	Admin	Ref.
Ap, CRO	Hd1	ACV	Hd7	DEX	Hd2	-	-	-	-	[37]
-	-	ACV	Hd10	CSS	NA	ACV	-	-	-	[10]
CRO	Hd1	-	-	MP, Pred	Hd2	-	-	IVIG	Hd5	[38]
-	-	-	-	-	-	Sedatives	-	-	-	[40]
CRO, AMX	Hd1-2	ACV	Hd8	MP	Hd7	-	-	-	-	[41]
CRO, VAN	Hd1	-	-	DEX, Pred	Hd10	-	-	-	-	[42]
CRO	NA	ACV	Hd1	-	-	-	-	-	-	[54]
-	-	-	-	-	-	ACV	-	-	-	[20]
CRO, VAN, MET, DOX	Hd1-Hd2	-	-	MP	Hd8	PHT, LEV, VAL, pentobarbital	Hd1, Hd4, Hd9	IVIG	Hd8	[44]
CRO, VAN, DOX	Hd4	-	-	-	-	Pentobarbital	Hd6	-	-	[46]
CRO, VAN	Hd1	ACV	NA	DEX	Hd1	-	-	-	-	[47]
CRO, Ap, DOX	NA	ACV	Hd1	-	-	-	-	-	-	[50]
-	-	ACV	Hd7	-	-	-	-	-	-	[25]
CRO, Ap, VAN	Hd1	ACV	Hd10	-	-	-	-	-	-	[51]
-	-	-	-	MP, Pred	-	-	-	IVIG	-	[52]
CRO, Ap, VAN	Hd1	ACV	Hd1	MP	Hd1	LEV	-	IVIG	-	[108]
CRO, AP, VAN	Hd1	ACV	Hd1	-	-	LEV, VAL, LCM	Hd4	IVIG	Hd7	[55]
CRO, VAN DOX	Hd1	ACV	Hd3	DEX	Hd3	LEV	-	-	-	[56]
-	-	-	-	-	-	LEV, LRZ	Hd1	-	-	[60]
Antibiotics	Hd1-2	-	-	MP	Hd5-10	LEV, phenytoin, LRZ	Hd1,Hd4	IVIG	Hd5	[58]
VAN, FEP	Hd1	-	-	MP, Pred	NA	LRZ, LEV	Hd1	IVIG	NA	[62]
CRO, Ap, VAN, DOX, PTZ	Hd1-2	ACV	Hd1	-	-	-	-	-	-	[61]

Abbreviations: ACV—acyclovir; AMX—amoxicillin; ACV—anticonvulsant; Ap—ampicillin; CSS—corticosteroids; CRO—ceftriaxone; DEX—dexamethasone; DOX—doxycycline; FEP—cefepime; IVIG—intravenous immunoglobulin; LCM—lacosamide; LEV—levetiracetam; LRZ—lorazepam; MET—metronidazole; MP—methlprednisone; NA—not available; PHT—phenytoin; Pred—prednisone; PTZ—piperacillin/tazobactam; VAN—vancomycin; VAL—valproic acid.

## Data Availability

All data are available within the manuscript or published references.

## Abbreviations

The following abbreviations are used in this manuscript:

CBC	Complete blood count
CDC	Centers for Disease Control and Prevention
CF	Complement fixation test
CHIKV	Chikungunya virus
CLIA	Clinical Laboratory Improvements Amendments
CSF	Cerebrospinal fluid
CT	Computed tomography
DENV	Dengue virus
E1	Envelope protein 1
E2	Envelope protein 2
E3	Envelope protein 3
EEE	Eastern equine encephalitis
EEG	Electroencephalogram
ELISA	Enzyme-linked immunosorbent assay
EEEV	Eastern equine encephalitis virus
FDA	Food and Drug Administration
FDG-PET	Fluorodeoxyglucose positron emission tomography
HI	Hemagglutination inhibition test
HSE	Herpes simplex encephalitis
IgM	Immunoglobulin M
IVIG	Intravenous immunoglobulin
MADV	Madriaga virus
MIA	Microsphere immunoassay
mNGs	Metagenomics next-generation sequencing
MRI	Magnetic resonance imaging
MVA-BN-WEV	Modified vaccinia Ankara, Bavarian Nordic, Western/Eastern/Venezuelan equine encephalitis virus multivalent vaccine candidate
NGS	Next-generation sequencing
nsP1	Non-structural protein 1
nsP2	Non-structural protein 2
nsP3	Non-structural protein 3
nsP4	Non-structural protein 4
NTPase	Nucleotide Triphosphatase
NYSDOH	New York State Department of Health
ORF	Open reading frame
PCR	Polymerase chain reaction
PRNT	Plaque-reduction neutralization test.
RNA	Ribonucleic acid
RTPase	RNA 5′ Triphosphatase
RT-PCR	Reverse transcription polymerase chain reaction
T2-FLAIR	T2-weighted fluid-attenuated inversion recovery
VEEV	Venezuelan equine encephalitis virus
VLDLR	Very low-density lipoprotein receptor
WBC	White blood cells
WEEV	Western equine encephalitis virus
ZIKV	Zika virus
